# Reaction of Amino-Terminated PAMAM Dendrimers with Carbon Dioxide in Aqueous and Methanol Solutions

**DOI:** 10.3390/molecules27020540

**Published:** 2022-01-15

**Authors:** Beijun Cheng, Angel E. Kaifer

**Affiliations:** 1 Department of Chemistry, University of Miami, Coral Gables, FL 33124, USA; b.cheng1@umiami.edu; 2 School of Chemistry and Chemical Engineering, Qilu University of Technology (Shandong Academy of Sciences), Jinan 250353, China

**Keywords:** carbon dioxide, PAMAM dendrimers, polyamines, carbamate formation

## Abstract

Polyamines have been used as active materials to capture carbon dioxide gas based on its well-known reaction with amines to form carbamates. This work investigates the reactions between three amino-terminated poly(amidoamine) (PAMAM) dendrimers (G1, G3 and G5) and CO_2_(g) in aqueous (D_2_O) and methanolic (CD_3_OD) solutions. The reactions were monitored using ^1^H NMR spectroscopy, and yielded dendrimers with a combination of terminal carbamate and terminal ammonium groups. In aqueous media the reaction was complicated by the generation of soluble carbonate and bicarbonate ions. The reaction was cleaner in CD_3_OD, where the larger G5 dendrimer solution formed a gel upon exposure to CO_2_(g). All reactions were reversible, and the trapped CO_2_ could be released by treatment with N_2_(g) and mild heating. These results highlight the importance of the polyamine dendrimer size in terms of driving changes to the solution’s physical properties (viscosity, gel formation) generated by exposure to CO_2_(g).

## 1. Introduction

**Scheme 1:**
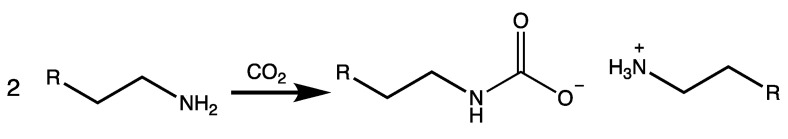
Reaction of CO_2_ with amines.

Carbon dioxide reacts with amines to form carbamic acid [1]. In the presence of excess amine, the acid becomes deprotonated, leading to a mixture of carbamates and protonated amines that may undergo electrostatic interactions, whose strength may depend on reactant concentrations and solvent nature, among other factors (Scheme 1). This reaction has been used in supramolecular systems [2,3] and is potentially useful to remove carbon dioxide gas from industrial emissions and, perhaps, for CO_2_ absorption as an atmospheric carbon capture and storage (CCS) method [4].

**Figure 1:**
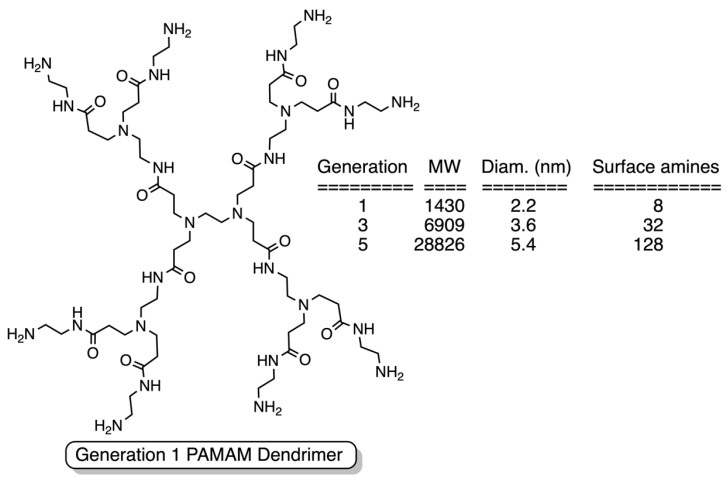
Structures of PAMAM dendrimers.

Our group’s past research [5,6] on the family of highly branched polymers known as dendrimers led us to consider the possibility of using amino-terminated dendrimers as active materials for CO_2_ capture. In fact, the commercially available poly(amidoamine) (PAMAM) dendrimers [7] have already been used within polymeric membranes for this purpose [8]. We were specifically interested in the corresponding carbamate-formation reaction in pure solvents, with a particular focus on the possibility of gel formation driven by the attractive electrostatic forces between the resulting carbamate and ammonium ionic groups on the dendrimer surfaces. Therefore, we focused our attention on the reactions of first (G1), third (G3) and fifth (G5) generation amino-terminated PAMAM dendrimers (see Figure 1) with CO_2_ gas in two polar solvents, water and methanol. These specific dendrimers were selected because of their commercial availability and the considerable molecular size difference between G1 and G5, while G3 was an intermediate case. From our experimental results, we conclude that the reaction takes place readily under mild conditions and can be easily reversed, that is, the reacted (trapped) CO_2_ can be released on demand. Furthermore, gel formation was detected with the largest G5 dendrimer, while an increase in solution viscosity was generally observed.

## 2. Results and Discussion

The ethylenediamine-core PAMAM dendrimers were obtained as concentrated solutions in methanol. We carried out our experiments either in deuterated water (D_2_O) or deuterated methanol (CD_3_OD) solutions. Carbon dioxide gas was bubbled though the dendrimer-containing solutions and the reaction was qualitatively monitored by recording the ^1^H NMR spectra as a function of time.

**Figure 2:**
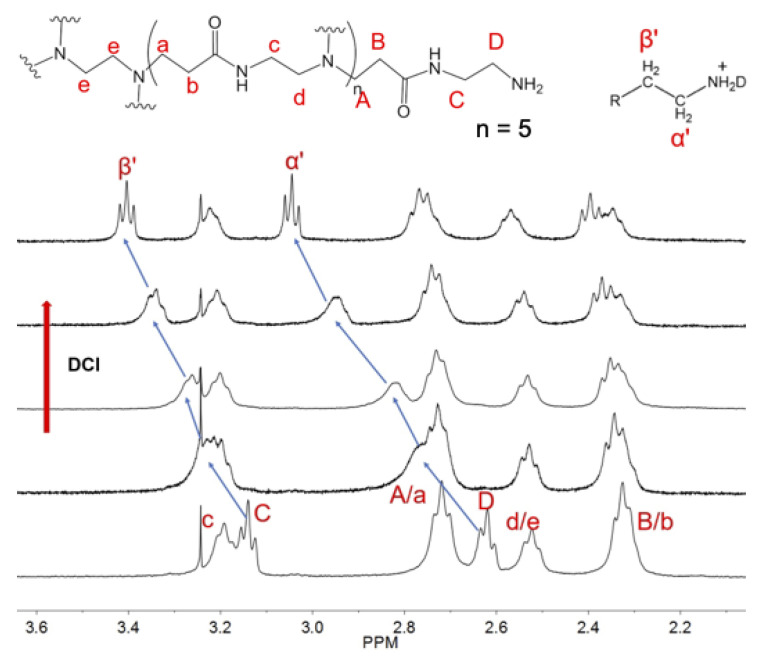
^1^H NMR spectra (400 MHz, D_2_O) of G5 dendrimer upon successive additions of DCl. The terminal methylene protons of the ammonium branches are labeled β′ and α’ (see upper right corner).

The NMR spectra of PAMAM dendrimers have been investigated before and we followed the findings of previous work [9,10] to assign the observed ^1^H NMR resonances in our experiments. The corresponding NMR spectra recorded in D_2_O solution are shown in the Supplementary Materials. Since the protonation of some of the terminal amine groups is expected to occur upon reaction with CO_2_, we first treated dendrimer D_2_O solutions with deuterium chloride (DCl). Figure 2 shows typical NMR spectra recorded upon acid additions to the fifth generation (G5) PAMAM dendrimer.

Clearly, the proton signals for the two methylenes (labeled C and D) adjacent to each of the terminal amino groups are the most affected by the protonation and shift smoothly downfield as the peripheral -NH_2_ groups become positively charged. The corresponding signals for the dendrimer’s protonated ammonium termini (–CH_2_CH_2_ NH_2_D^+^) are labeled as β’ and α’ in the figure. Further additions of DCl may lead to the protonation of internal tertiary amine groups, causing other shifts in the internal proton signals [11], but we did not monitor those changes as they are out of the scope of this work.

**Figure 3:**
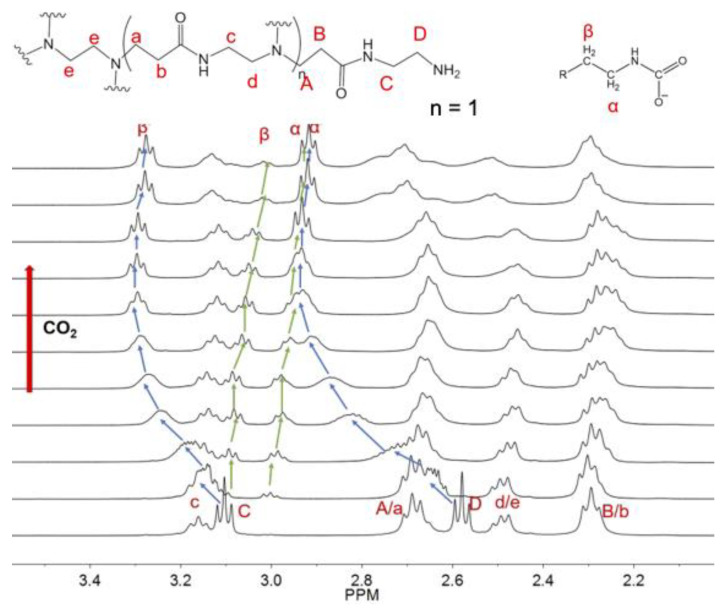
^1^H NMR spectra (400 MHz, D_2_O) of G1 dendrimer (0.139 M) upon continuous addition of CO_2_(g) to the solution. The terminal methylene protons of the ammonium branches are labeled β’ and α’ and those of the carbamate branches are labeled β and α (see upper right corner). The blue arrows follow the development of ammonium branch signals while green arrows follow the development of carbamate branches.

To follow the reaction with CO_2_, we bubbled this gas through the dendrimer solution, maintained at a temperature of 25 °C and under atmospheric pressure. The changes observed in the ^1^H NMR spectra were similar for all three generation PAMAM dendrimers (G1, G3 and G5). Representative spectra for G1 are shown in Figure 3.

As CO_2_(g) is bubbled through the solution, the terminal methylene proton signals of the NH_2_-terminated dendrimer branches decrease in intensity while new signals for the terminal methylene protons (β’ and α’) of the NH_3_^+^-terminated branches and the carbamate terminated branches (β and α) appear at approximately similar rates. However, further addition of CO_2_ leads to the conversion of some of the carbamate branches to ammonium branches, probably resulting from the carbonic acid formed in the solution, which destroys the carbamate. At higher concentrations of CO_2_, the solution becomes acidic, leading to the protonation of the dendrimer’s internal tertiary nitrogens, which affects the chemical shift of the neighboring proton signals. Similar data were collected with D_2_O solutions of the G3 and G5 dendrimers. The highest concentrations used in these experiments were 0.16, 0.10 and 0.011 M for the G1, G3 and G5 dendrimers, respectively. Even in the initial stages of these experiments, when the relative amounts of ammonium and carbamate branches were similar, we did not observe any significant increases in the solution viscosities, as a result of electrostatic forces (carbamate-ammonium) among dendrimers. This can be rationalized by considering the high polarity of the solvent, which effectively solvates the dendrimer surface charges, shielding the ion-ion electrostatic interactions. Furthermore, the carbamate formation reaction is complicated in aqueous media by the formation of carbonic acid and bicarbonate ions in the solution, affecting the overall stability of the carbamate-terminated branches on the dendrimer surfaces. For these reasons, we decided to carry out the remaining experiments in deuterated methanol solutions.

**Figure 4:**
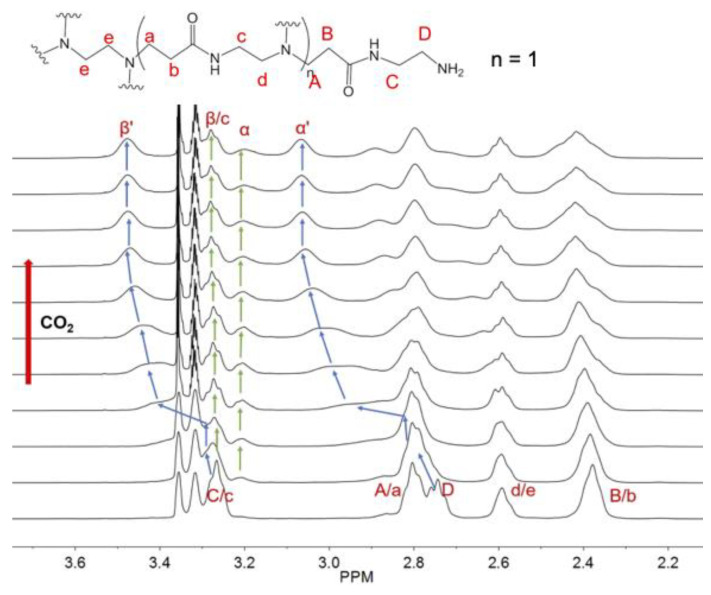
^1^H NMR spectra (400 MHz, CD_3_OD) of G1 dendrimer (0.02 M) upon addition of CO_2_(g) to the solution. As in the two previous figures, the terminal methylene protons of the ammonium branches are labeled β’ and α’ and those of the carbamate branches are labeled β and α. The blue arrows follow the development of ammonium branch signals while green arrows follow the development of carbamate branches.

The ^1^H NMR spectra of the dendrimers in CD_3_OD solutions are similar to those recorded in D_2_O (see Supplementary Materials). Initially, we carried out a series of experiments, in which the dendrimer concentration was fixed at 0.02 M. However, under these conditions, the initial concentrations of surface amine groups increase substantially with the generation of the dendrimer. In a second and more interesting series of experiments, we kept the initial concentration of surface amine groups constant at 2.56 M, which required using dendrimer concentrations of 0.32, 0.08 and 0.02 M for G1, G3 and G5, respectively. Figure 4 shows the NMR data for a 0.02 M CD_3_OD solution of G1. As it was the case in D_2_O solution, carbamate and ammonium branches seem to form simultaneously, but no carbamate conversion to ammonium was observed here. Excess of CO_2_(g) eventually leads to the protonation of internal tertiary nitrogens with the already mentioned effects on the neighboring proton signals. No gel formation was observed during any stages of this reaction.

**Figure 5:**
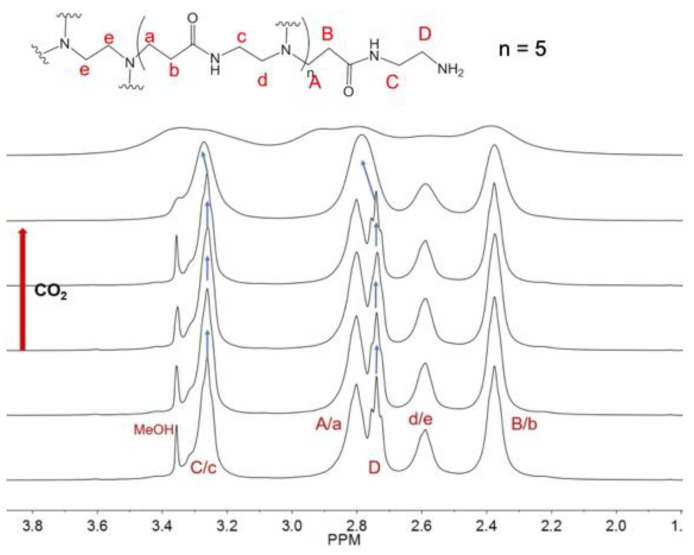
^1^H NMR spectra (400 MHz, CD_3_OD) of G5 dendrimer (0.02 M) upon addition of CO_2_(g) to the solution. Blue arrows are intended to guide the eye.

Bubbling of CO_2_(g) though a 0.02 M G5 solution in CD_3_OD leads, however, to different results. Figure 5 shows the recorded ^1^H NMR spectra. The signals are quite broadened from the beginning, thus diminishing our ability to follow the chemical changes associated with the addition of CO_2_(g). The signal broadening increases to a point at which little information can be obtained. At the same time, we observed that the solution becomes increasingly viscous as the CO_2_(g) bubbling continues and gel formation takes place. We believe that the signal broadening observed in this case is due to the large molecular mass (~29 KDaltons) and size (5.4 nm diameter) of this dendrimer, which limits its tumbling rate in the solution leading to NMR signal broadening. Furthermore, as CO_2_(g) is added, the NH_2_-terminated dendrimer branches are replaced by carbamate and ammonium terminated branches, which can give rise to considerable ion-ion interactions among dendrimers, leading to gel formation and further broadening of the NMR resonances. It is interesting that the NMR spectroscopic data obtained with the G3 dendrimer (Figure S10) under the same conditions is intermediate between what we observed with G1 and G5, with considerable signal broadening, increased solution viscosity, but without reaching full gel formation. Gel formation driven by CO_2_ reacting with the G5 dendrimer and the viscosity changes associated with the reaction with the other two dendrimers can be best visualized in video files included within the Supplementary Materials.

**Figure 6:**
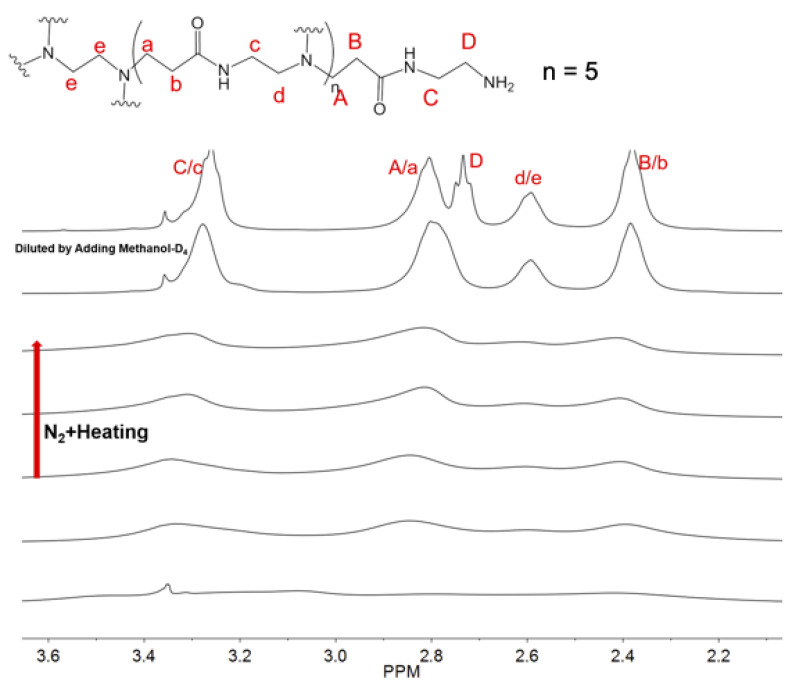
^1^H NMR spectra (400 MHz, CD_3_OD) of G5 dendrimer (0.02 M) upon addition of N_2_(g) to the solution under heating. The initial spectra (bottom) are extremely broadened due to gel formation. Addition of CD_3_OD at the end was necessary to replenish lost solvent due to evaporation.

A key question that we wanted to address was the reversibility of these processes. Is it possible to remove the carbon dioxide trapped as carbamate on the dendrimer surface and return the dendrimer to its original composition? To answer this question, we bubbled pure nitrogen gas through the solutions, while increasing the temperature to accelerate the possible CO_2_(g) release. In all cases, we recovered the intact starting material (G1, G3 or G5), as evidenced by the final NMR spectrum recorded after extensive N_2_(g) bubbling under heating (see Figure 6). However, this process was increasingly slower for the larger dendrimers and a considerable level of solvent evaporation took place, which required replacing some of the CD_3_OD solvent to record the final spectrum. In the case of G5, our data allowed us to conclude that gel formation is driven by reaction with carbon dioxide and can be reversed by a combination of heating and N_2_(g).

At this point, it is illustrative to perform some straightforward calculations to understand the differences among 0.02 M solutions of these three PAMAM dendrimers (G1, G3 and G5). If we consider the dendrimers as spheres with the diameters provided in Figure 1, we can easily calculate that they will, respectively occupy 6.71, 29.4 and 99.3% of the total volume in their 0.02 M solutions. We assumed that the dendrimers have spherical shapes and calculated their molecular volumes (4πr^3^/3) using the radii taken from the diameters listed in Figure 1. Then, since the dendrimer concentration is 0.02 M, we considered a total solution volume of 1.00 dm^3^ and computed the volume occupied by the dendrimers by multiplying the molecular dendrimer volume by (0.02 × 6.022 × 10^23^). The resulting value was taken as the partial volume occupied by the dendrimer and compared to the total solution volume of 1.00 dm^3^ (1.00 L). Of course, the accuracy of this calculation improves with dendrimer generation, as dendrimers resemble spheres more accurately as their molecular mass increases. The smaller dendrimers, G1 and G3, have more open structures, which facilitate the permeation of solvent molecules to their interior crevices and cavities. However, after acknowledging the approximations involved in these calculations, it is clear that their accuracy would be best for G5 compared to the smaller dendrimer analogs. Therefore, the fact that about 99% of the volume is occupied by the G5 dendrimer in its 0.02 M solution, clearly indicates that there are few CD3OD molecules available to shield the electrostatic interactions between carbamate and ammonium branches, thus explaining the formation of gels upon exposure and reaction with carbon dioxide. The much lower volume fractions of the 0.02 M solutions occupied by either G3 or G1, leaves much higher volume fractions to be filled by solvent (CD3OD) molecules, which are thus capable to shield the dendrimer-dendrimer electrostatic forces, thus preventing the formation of gels.

## 3. Materials and Methods

The PAMAM dendrimers were purchased as methanolic solutions from Dendritech (Midland, Michigan) and used after solvent evaporation and vacuum drying to avoid large signals from the methanol protons on the NMR spectra. Deuterated solvents for NMR experiments (D_2_O and CD_3_OD) were obtained from Cambridge Isotope Laboratories. The dendrimer solutions were placed in 5 mm NMR tubes and carbon dioxide gas was bubbled through them using narrow (1 mm dia.) Teflon tubing. The reaction with CO_2_(g) was conducted at 25 °C and atmospheric pressure. Carbon dioxide purging was interrupted at various time intervals to record the ^1^H NMR spectrum of the sample. To investigate the release of the trapped CO_2_, the solutions previously treated with this gas were purged with N_2_(g) at a temperature of ca. 60 °C. Under these conditions, some of the solvent evaporated, which required replenishing the lost CD_3_OD before recording the final NMR spectra to confirm recovery of the starting dendrimer. All ^1^H NMR spectra were recorded on a Bruker (Billerica, Massachusetts) Avance spectrometer (operating at 400 MHz).

## 4. Conclusions

Overall, we conclude that the reaction of CO_2_(g) with amine-terminated PAMAM dendrimers leads to the anticipated conversion of the terminal amines to carbamates and ammonium groups. While this reaction was complicated in the aqueous (D_2_O) solution by the concomitant generation of free carbonate and bicarbonate ions, which can lead to the eventual decomposition of the terminal carbamates, it is much cleaner in the methanolic (CD_3_OD) solution, leading to considerable viscosity increases and/or gel formation. The latter was only clearly observed with the larger G5 dendrimer at an initial concentration of 0.02 M. In all cases, the CO_2_ trapped as carbamate on the dendrimer surface could be released by treatment with N_2_(g) and mild heating, showing that the reaction is reversible.

While dendrimers are too expensive to be proposed as active materials for CO_2_(g) capture, our results here illustrate the effects of dendrimer (polymer) size at bringing about physical property changes (viscosity, gel formation) that can be used to advantage in future schemes for carbon dioxide removal and capture from industrial emissions or directly from the atmosphere.

## Supplementary Materials

The following are available online, Scheme 1: Reaction of carbon dioxide with amines, Figures S1–S11: Additional NMR spectroscopic data, Videos S1–S3: Viscosity behavior of dendrimer solutions after reaction with carbon dioxide.
