# Supplementary material for: Reaction of Amino-Terminated PAMAM Dendrimers with Carbon Dioxide in Aqueous and Methanol Solutions
Source: Molecules. 2022 Jan 15;27(2):540. doi: 10.3390/molecules27020540 (PMC8777730; doi:10.3390/molecules27020540)
Supplement: Supplementary file 1 [file molecules-27-00540-s001.zip › molecules-1525392-supplementary.pdf]

SUPPLEMENTARY MATERIALS

# Reaction of amino-terminated PAMAM dendrimers with carbon dioxide in aqueous and methanol solutions

Beijun Cheng[1,2], and Angel E. Kaifer[1,*]

[1]Department of Chemistry, University of Miami, Coral Gables, FL 33124, USA
[2]Current address: School of Chemistry and Chemical Engineering, Qilu University of Technology (Shandong Academy of Sciences), Jinan, Shandong, 250353 China
*Correspondence: akaifer@miami.edu

TABLE OF CONTENTS

Page(s)
======

Reaction of carbon dioxide with amines ……………………………….. S2

$^{1}$H NMR spectra of dendrimers G1, G3 and G5 in $D_2O$ ……………… S2-S4

Spectral evolution under $CO_2$(g) for G3 and G5 in $D_2O$ …………….. S5-S6

$^{1}$H NMR spectra of dendrimers G1, G3 and G5 in $CD_3OD$ …………. S7-S9

Spectral evolution under $CO_2$(g) for G1 and G3 in $CD_3OD$ ………… S10-S11

Video files ………………………………………………………………… S12

Recovery of initial material in the case of dendrimer G3 ……………. S13

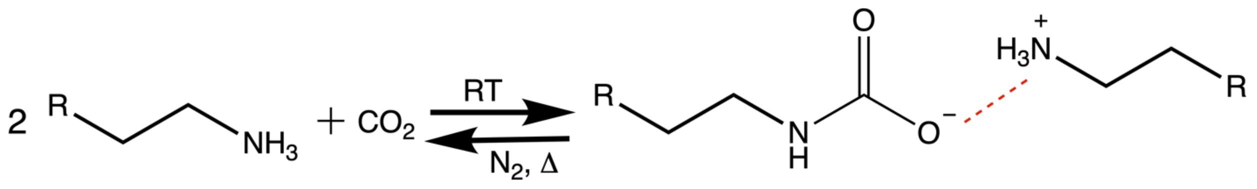


**Scheme S1**. Reaction of carbon dioxide with amines.

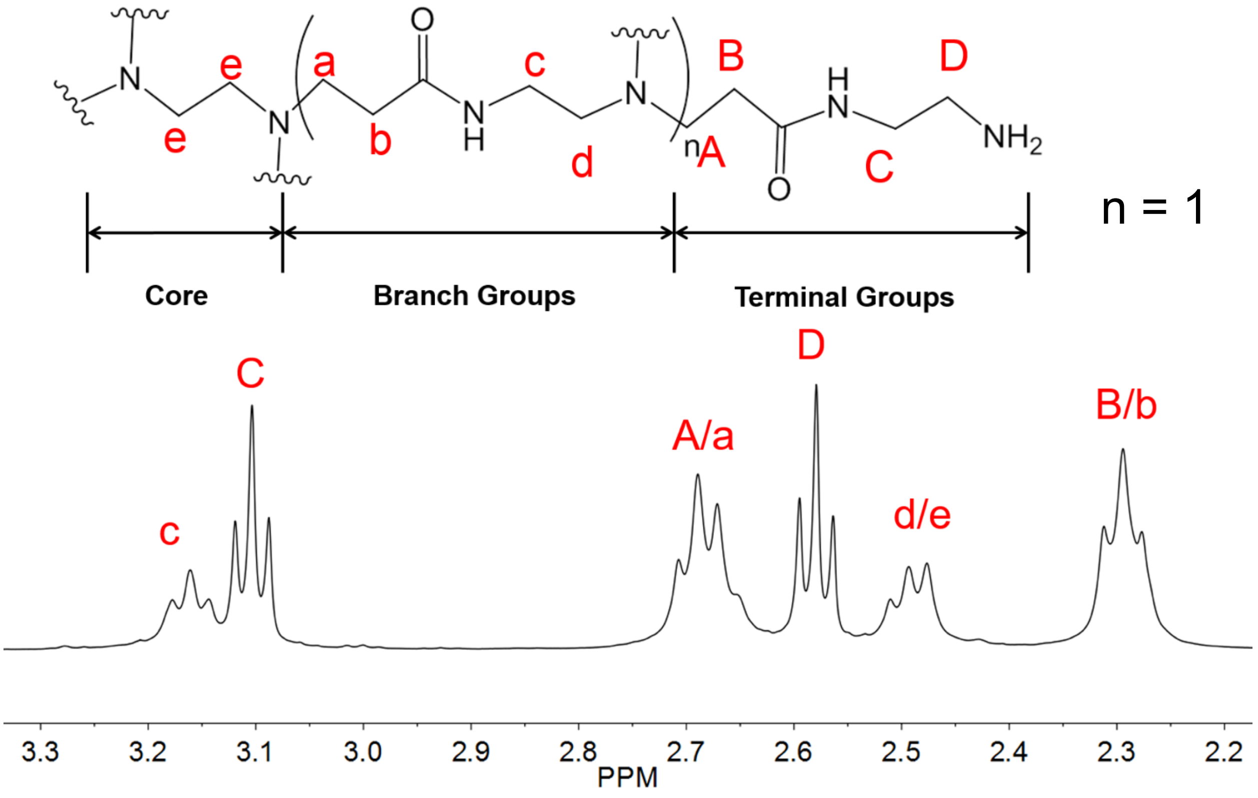


**Figure S1.** $^{1}$H NMR (400 MHz, $D_2O$) of first generation PAMAM dendrimer (G1).

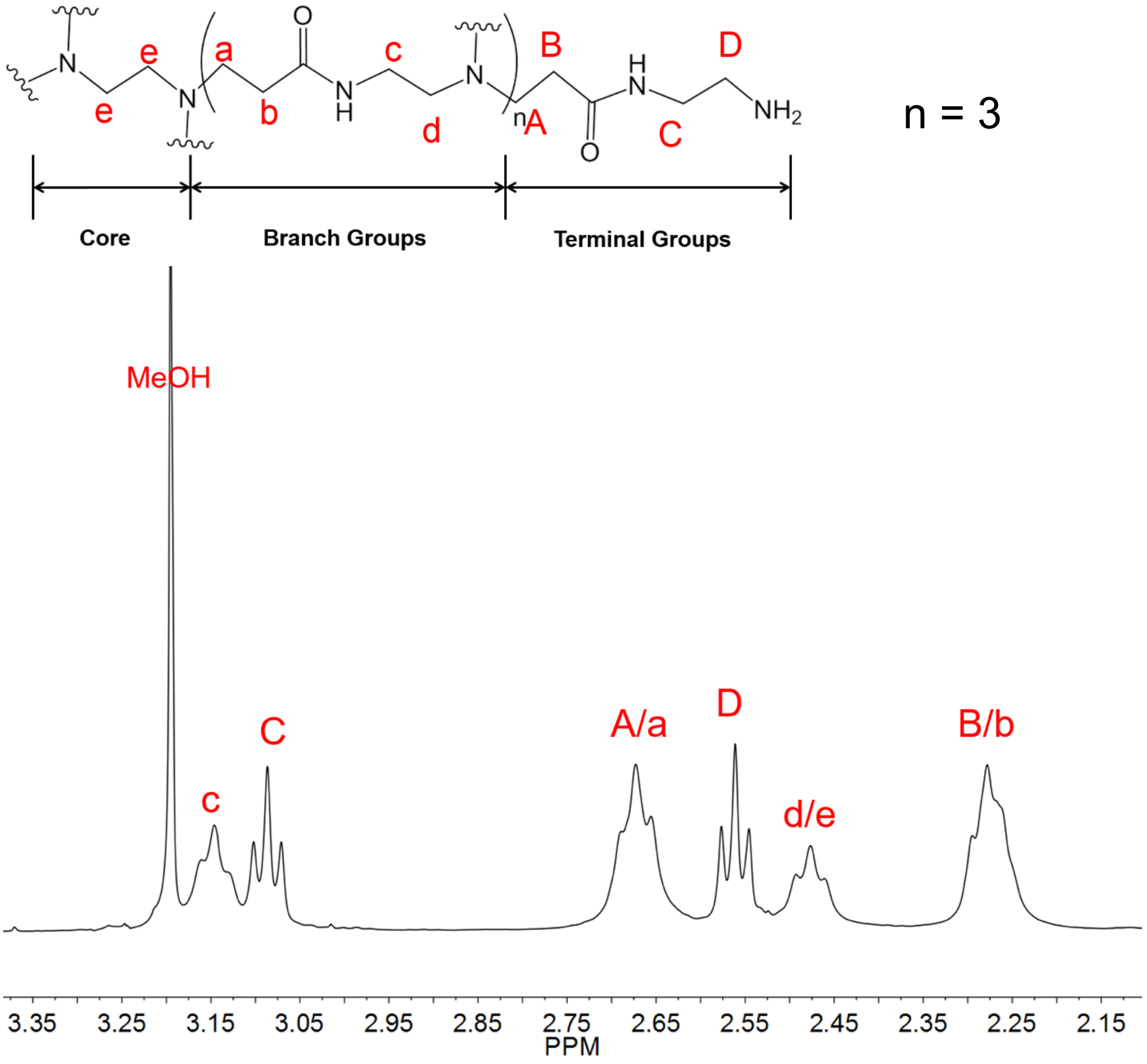


**Figure S2.** $^1$H NMR (400 MHz, $D_2O$) of third generation PAMAM dendrimer (G3).

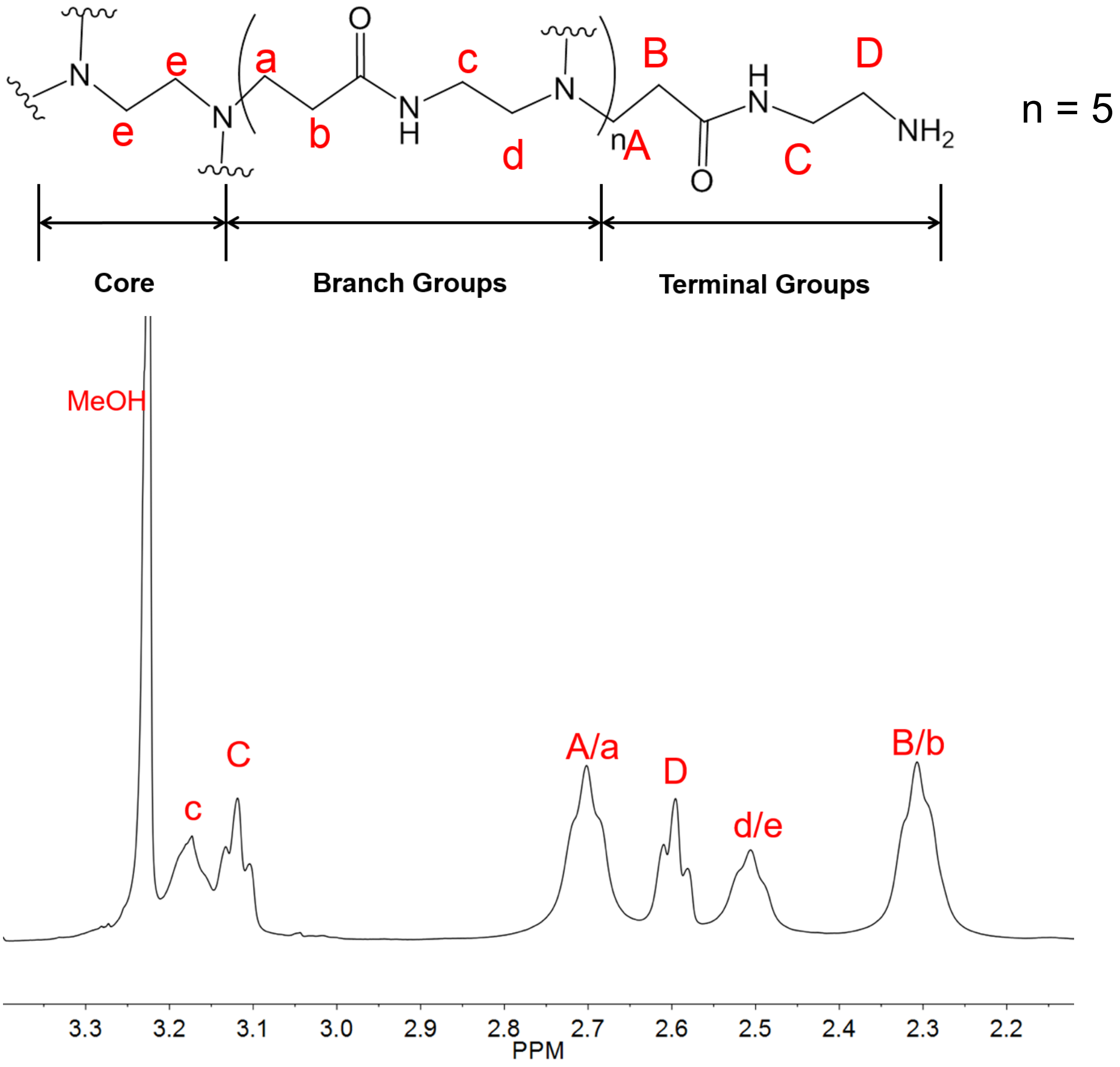


**Figure S3.** $^{1}$H NMR (400 MHz, $D_2O$) of fifth generation PAMAM dendrimer (G5).

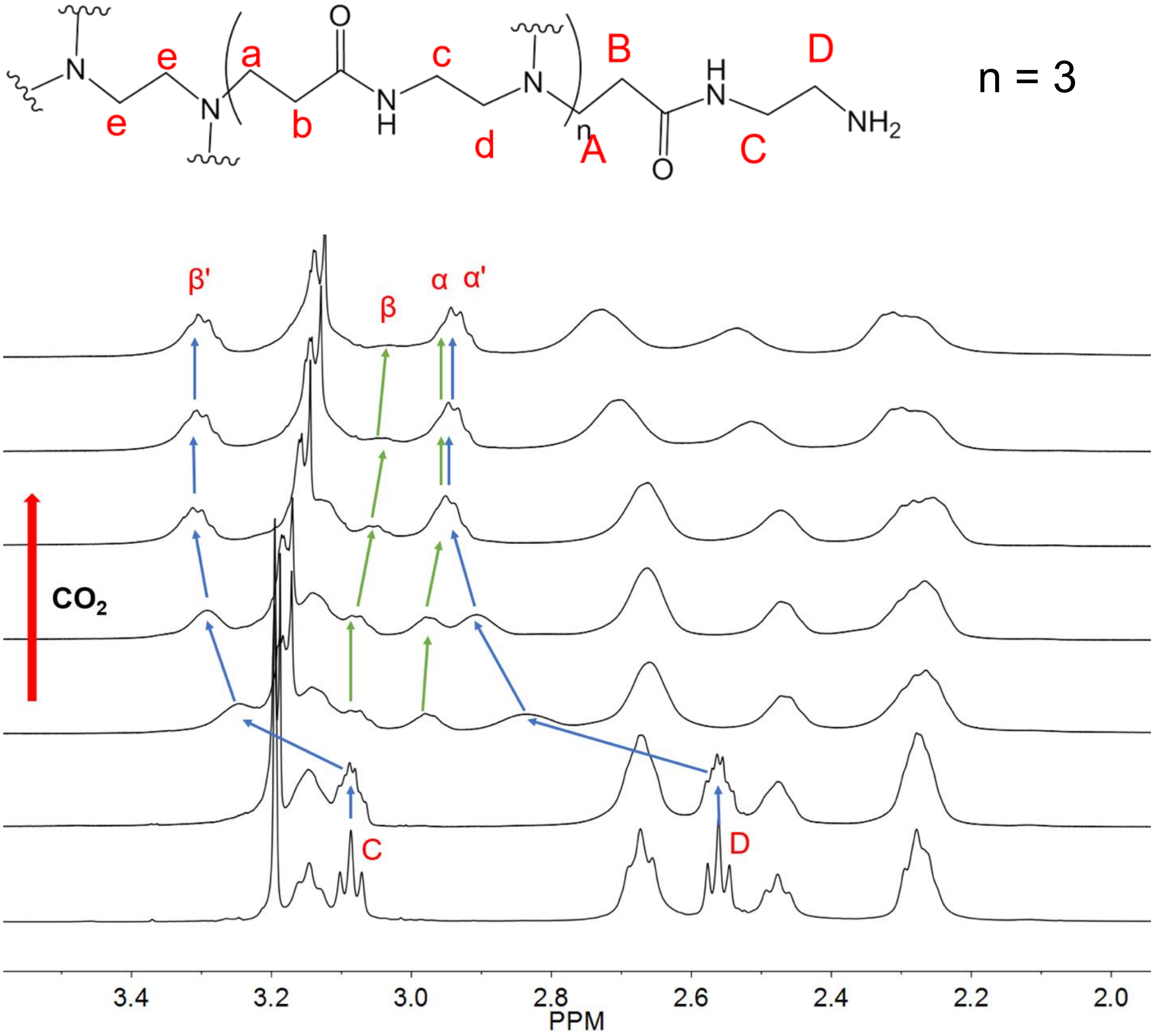


**Figure S4.** $^{1}$H NMR spectra (400 MHz, $D_2O$) of G3 dendrimer (0.026 M) upon addition of $CO_2$(g) to the solution. The terminal methylene protons of the ammonium branches are labeled β′ and α′ and those of the carbamate branches are labeled β and α.

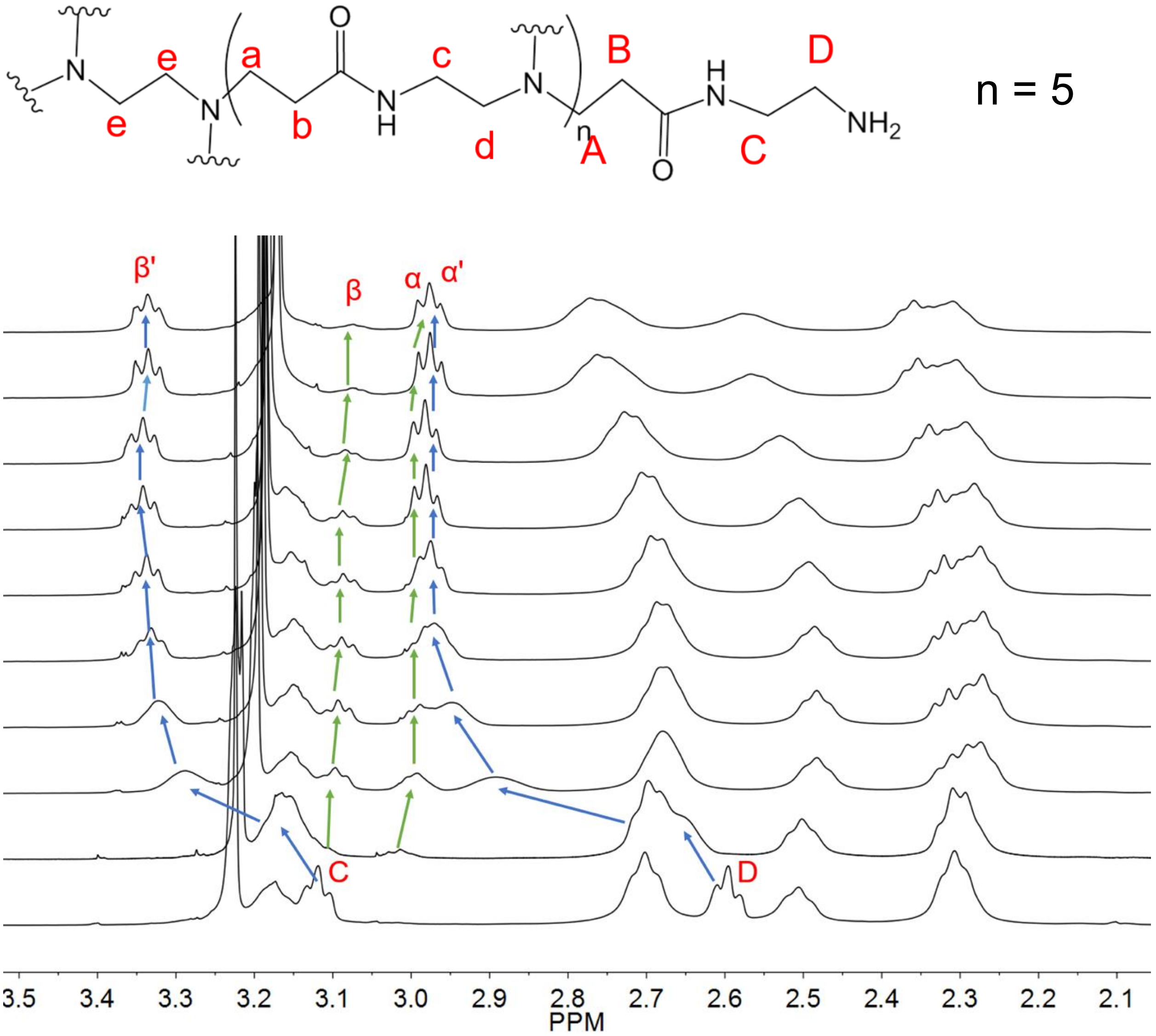


**Figure S5.** $^1$H NMR spectra (400 MHz, $D_2O$) of G5 dendrimer (0.009 M) upon addition of $CO_2$(g) to the solution. The terminal methylene protons of the ammonium branches are labeled β′ and α′ and those of the carbamate branches are labeled β and α.

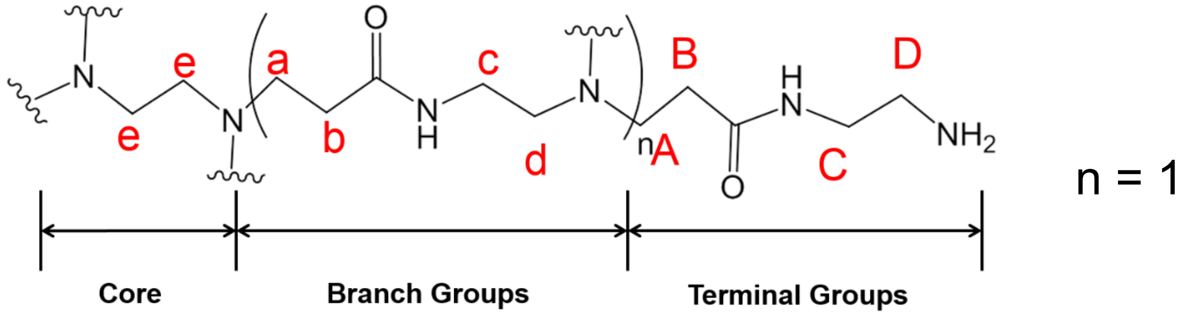


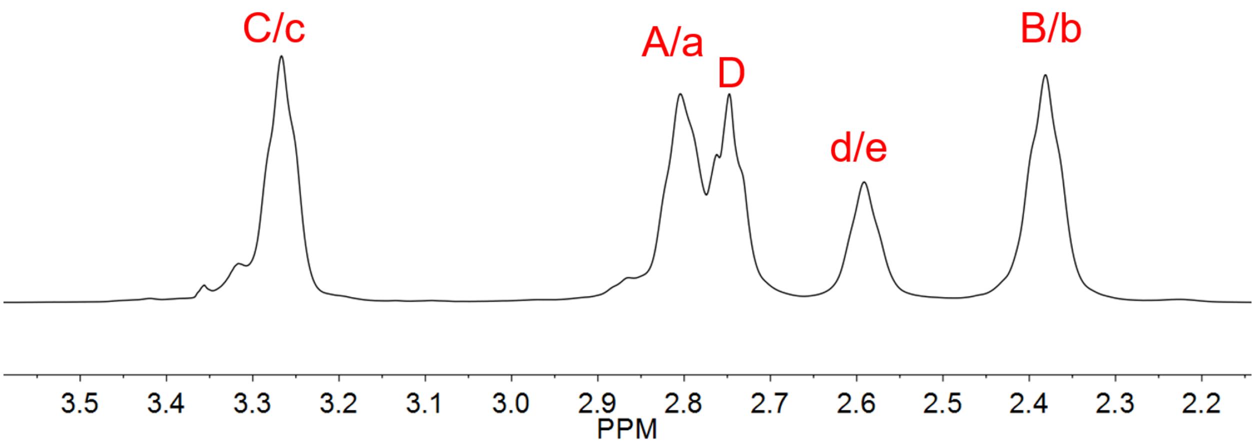


**Figure S6.** $^{1}$H NMR spectrum (400 MHz, $CD_3OD$) of first generation dendrimer (G1)

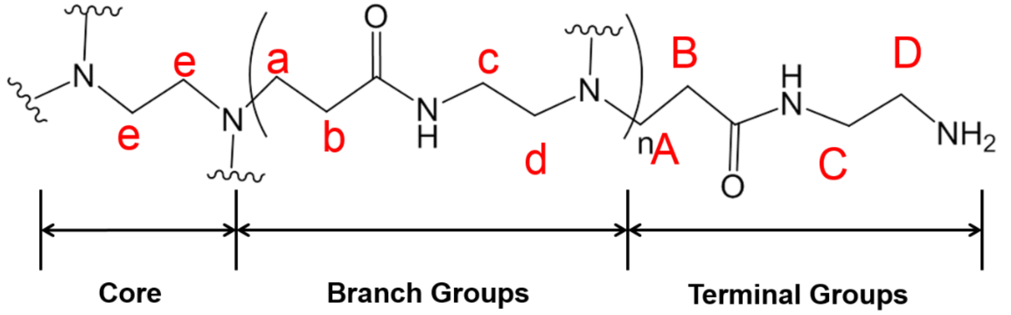


n = 3

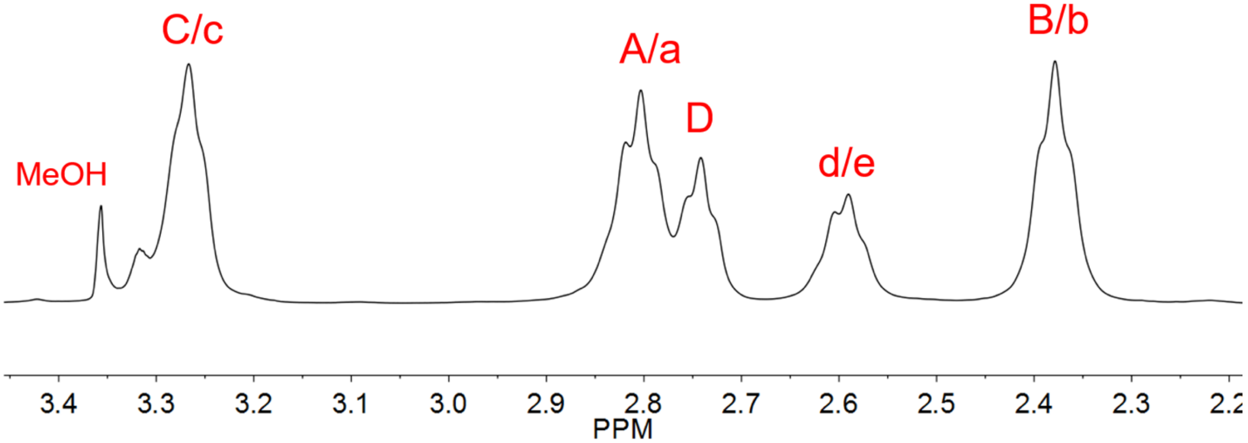


**Figure S7.** $^1$H NMR spectrum (400 MHz, $CD_3OD$) of third generation dendrimer (G3)

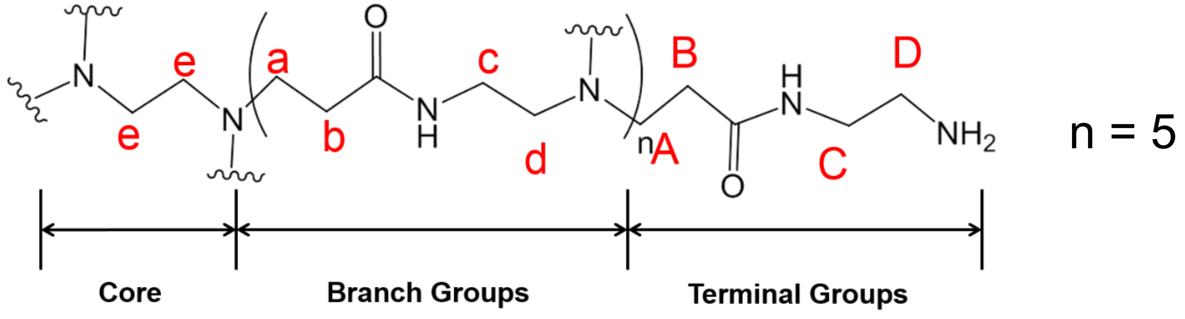


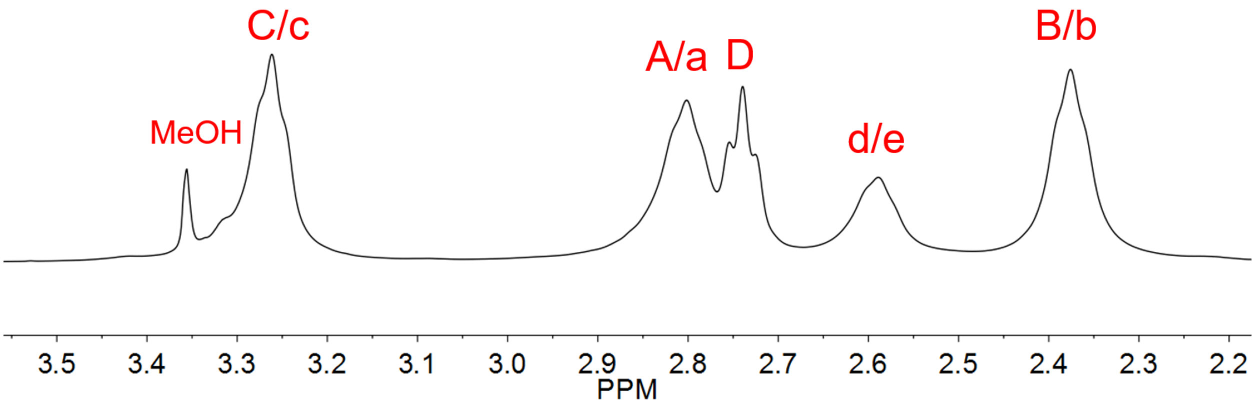


**Figure S8.** $^{1}$H NMR spectrum (400 MHz, $CD_3OD$) of fifth generation dendrimer (G5)

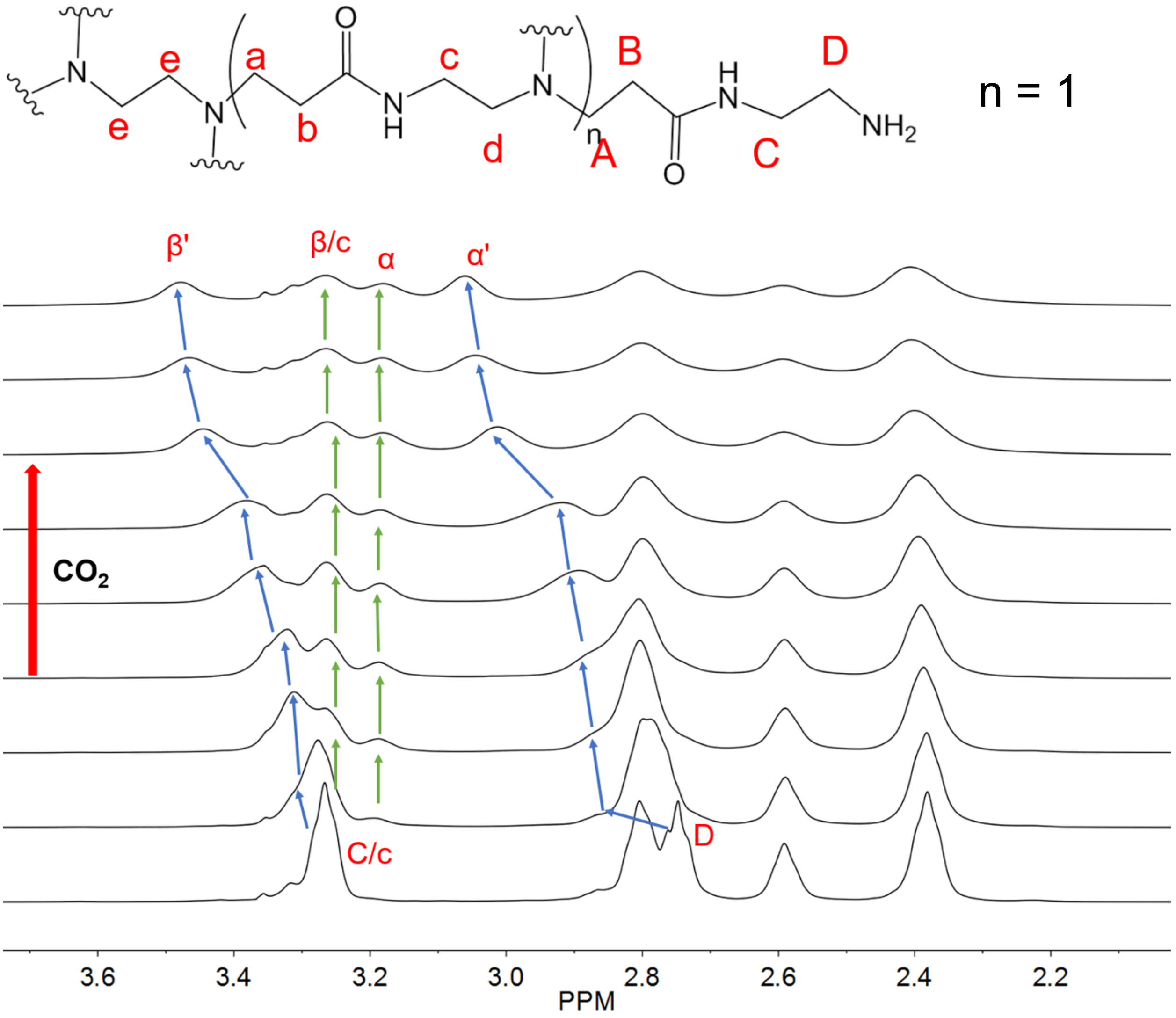


**Figure S9**. $^{1}$H NMR spectra (400 MHz, $CD_3OD$) of G1 dendrimer (0.32 M) upon addition of $CO_2$(g) to the solution. The terminal methylene protons of the ammonium branches are labeled β′ and α′ and those of the carbamate branches are labeled β and α.

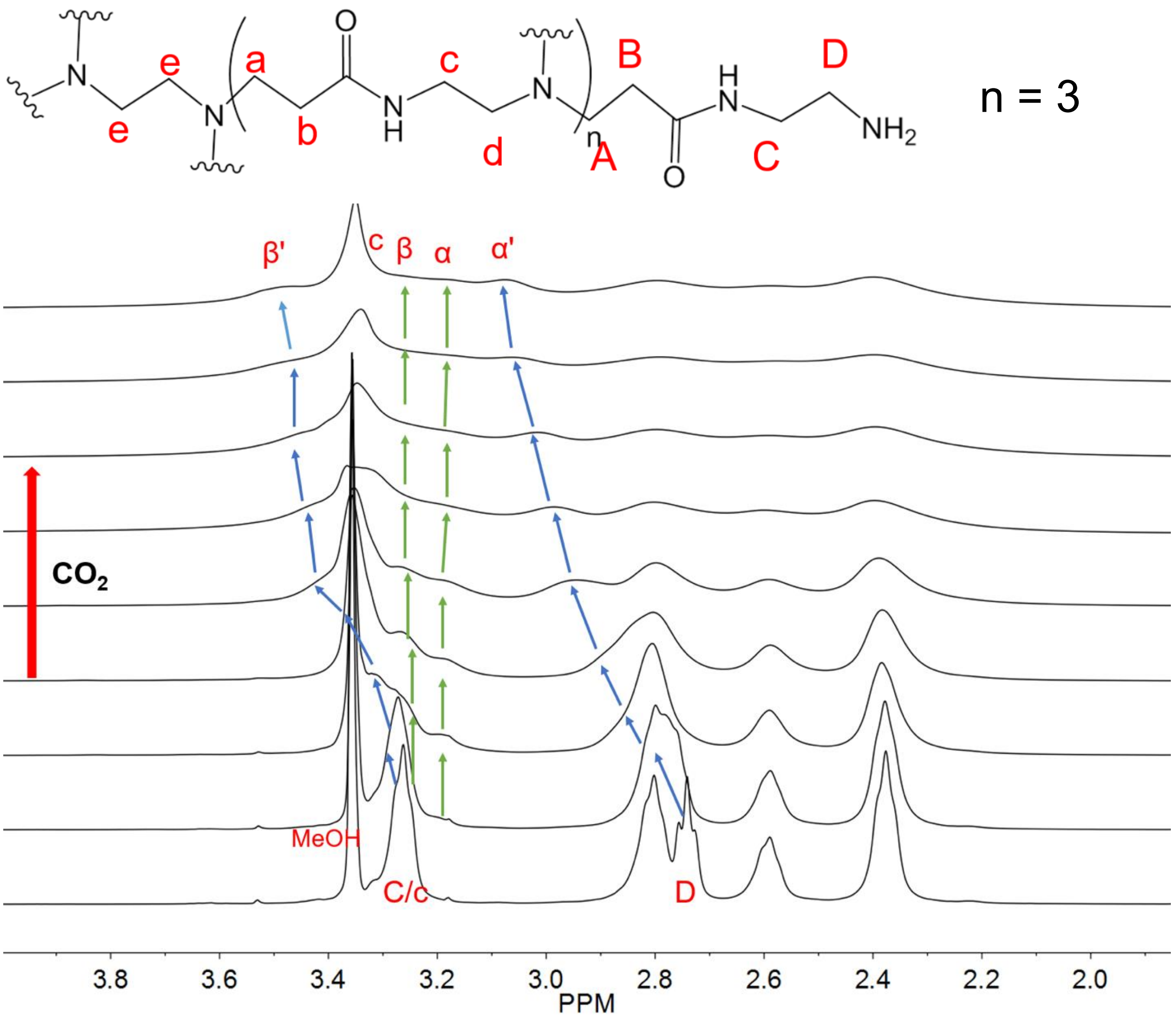


**Figure S10**. $^{1}$H NMR spectra (400 MHz, $CD_3OD$) of G3 dendrimer (0.080 M) upon addition of $CO_2$(g) to the solution. The terminal methylene protons of the ammonium branches are labeled β′ and α′ and those of the carbamate branches are labeled β

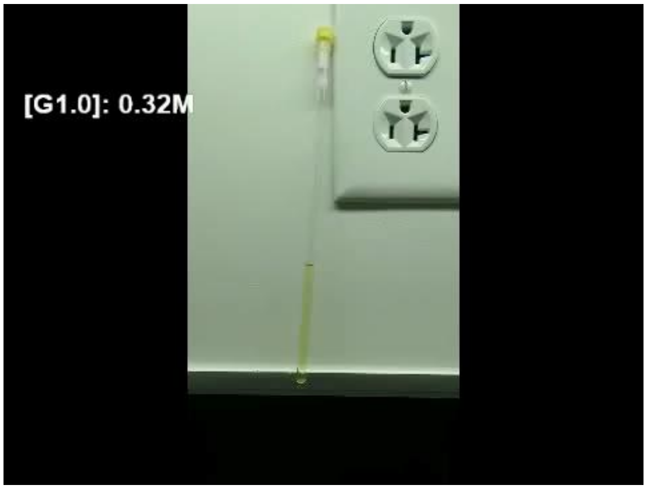


**Video S1.** Behavior of a 0.32 M methanolic solution of the G1 dendrimer after reaction with $CO_2$(g). (Double click on the file to play the video).

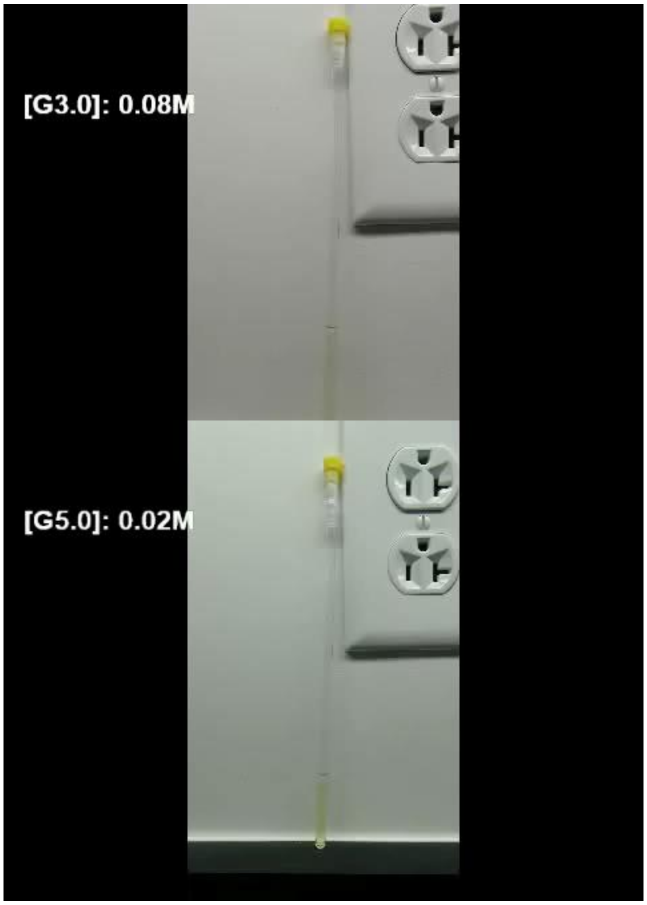


**Video S2.** Behavior of a 0.080 M methanolic solution of the G3 dendrimer after reaction with $CO_2$(g). (Double click on the file to play the video).

**Video S3.** Behavior of a 0.020 M methanolic solution of the G5 dendrimer after reaction with $CO_2$(g). (Double click on the file to play the video).

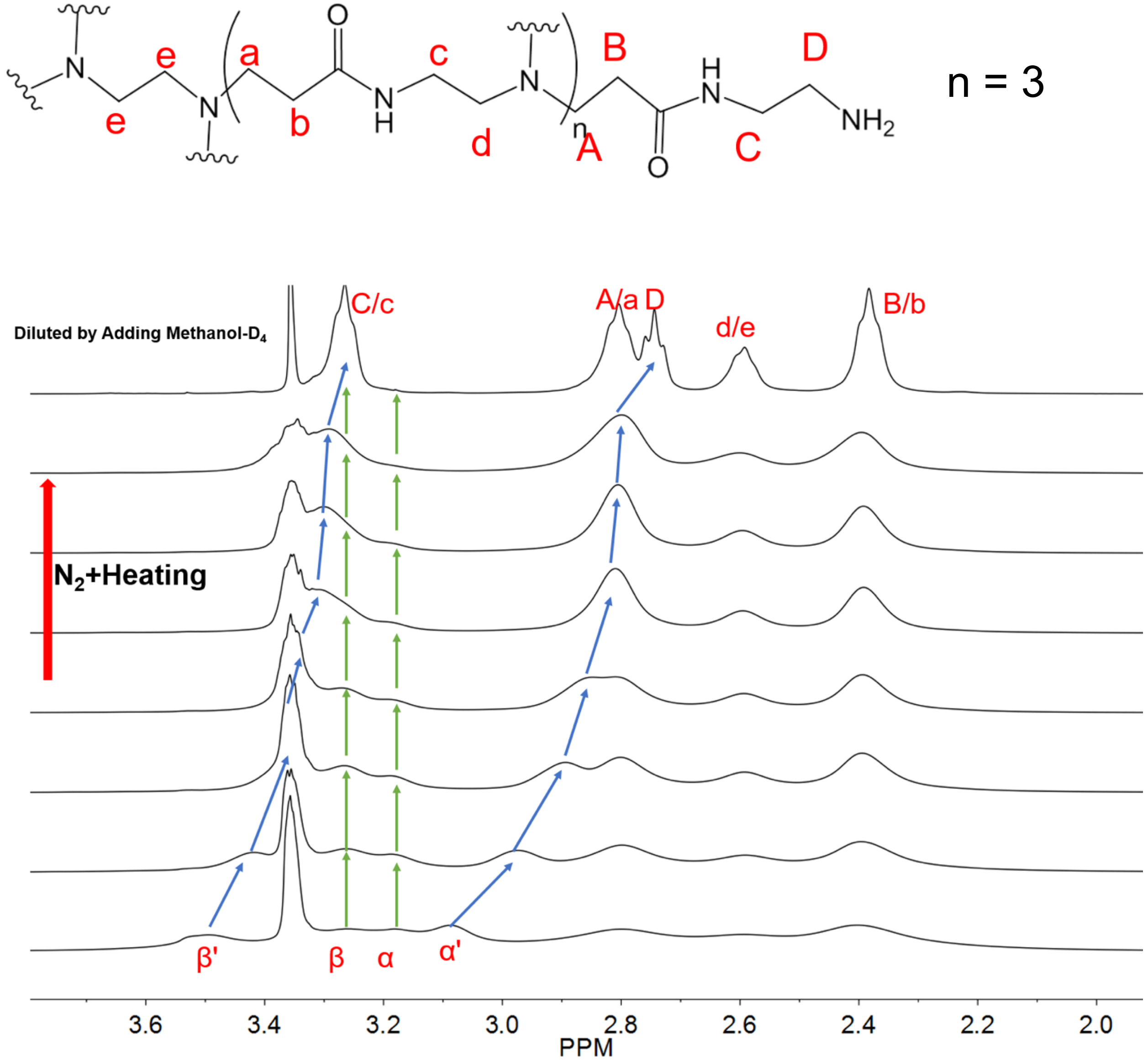


**Figure S11**. $^1$H NMR spectra (400 MHz, $CD_3OD$) showing the recovery by heating and purging with $N_2$(g) of the initial spectrum for G3 after its reaction with $CO_2$(g).
